# Xylan-Derived Light Conversion Nanocomposite Film

**DOI:** 10.3390/polym12081779

**Published:** 2020-08-09

**Authors:** Yunyi Yang, Yushuang Zhao, Yijie Hu, Xinwen Peng, Linxin Zhong

**Affiliations:** State Key Laboratory of Pulp and Paper Engineering, South China University of Technology, Guangzhou 510641, China; 201730440313@mail.scut.edu.cn (Y.Y.); 15574930182m0@sina.cn (Y.Z.); 201710104846@mail.scut.edu.cn (Y.H.); fexwpeng@scut.edu.cn (X.P.)

**Keywords:** hemicelluloses, xylan, biomass, hydrothermal, carbon dots

## Abstract

A new type of sustainable light conversion nanocomposite film was fabricated by using carboxymethyl xylan as matrix and xylan-derived carbon dots (CDs) as both light conversion regents and nano reinforcements. The results demonstrate that CDs can not only significantly enhance the mechanical strength of the nanocomposite film because of chemical reaction between CDs and carboxymethyl xylan, but also impart the film with excellent optical properties. With 1.92 wt% CDs, the tensile strength and elastic modulus of the film are increased by 114.3% and 90.7%, respectively. Moreover, the film has typical excitation and emission spectra, enabling the efficient absorption of UV and the conversion of UV to blue light. This xylan-derived light conversion nanocomposite film is expected to be used in agricultural planting and food packaging.

## 1. Introduction

Light conversion film is a kind of functional film that can change the wavelength of light [1], and shows great potential in the fields of agriculture [2], food industry [3], skin care [4], and so on. For example, red light and blue light can be absorbed by plants to facilitate chlorophyll synthesis, while ultra-violet (UV) components in sunlight cannot be utilized and will induce pathological changes of plants [2]. Thus, it is of great significance to use light conversion film to convert UV to blue light to protect plants and boost their growth. The UV absorption of dairy foods, such as milk products, can result in significant loss of valuable nutrients and discoloration, as well as formation of strong off-flavors [3]. Additionally, UV is also harmful to human skin and can cause erythema and skin cancel. Therefore, light conversion film is highly required to protect our foods and skin from UV radiation [5].

Light conversion film is composed of light conversion agent and matrix, where light conversion agent acts as a pivotal role [1]. Conventional light conversion agents are mostly organic dyes, such as triphenylacrylonitrile [6] and perylene diimides [7]. However, these organics are toxic and easily degraded under light [8]. On the other hand, most of inorganic photoactive materials, such as nano ZnO and TiO_2_, easily aggregate at a certain concentration, reducing the transparency of final films [9]. Carbon dots (CDs) are a new type of light conversion agents that can effectively absorb certain characteristic wavelength and allow electrons to transform from ground state to excited state. Meanwhile, a part of energy is released in the form of photons when transition from the lowest excited state to the ground state, and thus the whole process of sunlight conversion is completed [1]. CDs from renewable biomass [10], such as wheat straw [11], grass [12], protein [13,14], chitosan [15], cellulose fiber [16], lignin [17], and hemicelluloses [18], are especially attractive and become alternatives to conventional light conversion agents due to their high light conversion, sustainability, and low cost.

Conventional matrixes are mainly petrochemicals, such as polythene [19] and polyvinyl chloride [6], because of their excellent film formation, good mechanical properties, and transparency. However, the nonrenewability and nonbiodegradability of these synthetic polymers pose a great hazard to environment. Renewable biomass resources, including hemicelluloses [20], cellulose [21,22] and chitosan [23], are environmental-friendly, biodegradable, and biocompatible, making them attractive matrixes for light conversion films. Up to now, there has been a few composite films fabricated by combining biomass resources such as cellulose [24] or chitosan [25] and CDs for biomedicine application [24,25,26,27,28]. For example, Yu et al. [24] fabricated a CD-glucose oxidase/cellulose acetate film (L-cysteine and citric acid monohydrate as precursors to synthesize CDs) that exhibited high sensitivity when being used as an optical fiber glucose biosensor. Cuevas et al. [26] obtained a luminescent cellulose film with improved electrical conductivity and mechanical strength by incorporating different silicon-dot and CDs (from lactose) into cellulose matrix. However, light conversion film based on biomass-derived CDs and biomass matrix has rarely been investigated.

Hemicelluloses such as xylan or glucomannan are the secondly abundant polysaccharides in plants, accounting for 20–30 wt% in dry plants. However, the relatively low molecular weight of hemicelluloses usually leads to poor mechanical performances [29]. Chemical modification can improve the film-forming and mechanical performances of hemicelluloses [30]. However, up to now, hemicelluloses/CDs film has not been reported. Herein, a xylan-derived light conversion film was fabricated by using carboxymethyl xylan as matrix and xylan-derived CDs as light conversion regents and nano reinforcements. The interaction between matrix and CDs was revealed. The important roles of CDs on imparting the film with light conversion performances and enhancing the mechanical strength of film were investigated.

## 2. Materials and Methods 

### 2.1. Materials

Xylan (weight-average molecular weight *M*_w_ = 133,692), which was kindly supplied from Shandong Longlive Co., Ltd., contains 85.4% xylose, 6.3% arabinose, 1.5% galactose, 3.2% glucose, 2.2% galacturonic acid. Quinine sulfate dehydrate (99.0%, Biotech), sodium hydroxide (ACS, 97.0%), hydrochloric acid (36%~38%), sodium chloroacetate, acetic acid glacial were of analytical grade and bought from Aladdin (China). Polyvinyl alcohol (PVA) (*M*_w_: 30,000–70,000) was bought from Sigma-Aldrich. Water was ultrapure (R: 18.2 MΩ·cm).

### 2.2. Preparation of CDs

CDs were prepared according to our previous method [16]. Firstly, 1.5 g xylan was dispersed in ultrapure water (30 mL) by stirring before being transferred to stainless steel autoclaves (SLM-50). The hydrothermal reaction was carried out in a furnace under 600 rpm at 240 °C for 6 h. After cooling to room temperature, thoroughly washing with deionized water, and drying in a vacuum oven at 80 °C, hydrothermal carbon (HTC) was obtained. Then, 50 mg HTC was added into 50 mL Teflon-lined stainless-steel autoclave (SLM-50) with 25 mL 0.1 M NaOH. The chamber was sealed and O_2_ was blown into the autoclave to remove air. Afterwards, the autoclave (with 1 MPa O_2_) was heated at 160 °C for 1 h with stirring (1000 rpm). After naturally cooling to room temperature, a yellow suspension was obtained and neutralized by HCl. The mixture was filtered using 0.22 μm microporous film and the filtrate was dialyzed in a dialysis bag (retaining molecular weight = 500 Da) in deionized water for 5 days (replacing fresh water for 12 h). Finally, the suspension was freeze-dried to obtain pure CDs.

### 2.3. Preparation of Carboxymethylated Xylan (CX)

6.6 g xylan was suspended in 60 mL 20% ethanol solution by violent stirring. After heating the mixed solution to 30 °C, 32.88 mL 25% NaOH solution was slowly added and stirred for 30 min. 23 g sodium monochloroacetate was added to the mixed solution and heated to 65 °C for 2 h. Then, 32.88 mL 25% NaOH solution and 23 g sodium monochloroacetate was added slowly, and the reaction was processed for 1 h at 65 °C. The precipitate was subsequently filtered and washed thoroughly with 75% ethanol. Finally, CX was obtained by freeze-drying the precipitate.

### 2.4. The Degree of Substitution (DS) of Carboxyl Group in CX

The degree of substitution (DS) of carboxyl group in CX was measured by a conductivity method. 0.1 g CX was dissolved in 100 mL NaCl solution and the pH of the solution was adjusted to 4 by 0.1 M hydrochloric acid. After adding 0.01 M NaOH standard solution in nitrogen atmosphere, the change of conductivity was recorded. The DS of CX was calculated by Formulas (1) and (2):

(1) $$B=M\left(V_{2}-V_{1}\right)/m$$

(2) DS=0.132×B/(1−0.008×B)

where m is the weight of CX (g), M is the standard molar concentration of NaOH (mol/L), V_1_ is the volume of NaOH at the left isoelectric point (mL), V_2_ is the volume of NaOH at the right isoelectric point (mL), and B is the NaOH consumed per gram of CX (mmol/g). The DS of carboxyl group is 0.43.

### 2.5. Preparation of CX/PVA-CDs Composite Films

13 mL ultrapure water, 0.4 g CX and 0.1 g PVA were mixed homogeneously, followed by adding different contents of CDs (0, 0.48, 0.96, 1.44, and 1.92 wt%, based on the total mass of CX and PVA). The mixture was stirred at 80 °C for 1 h. After reaction, the mixture was cooled down to room temperature and then poured into a polyethylene Petri dish with a diameter of 9.0 cm. Composite films were obtained after drying the mixture at room temperature. The as-prepared composite films were named as CX/PVA, CX/PVA-CDs_0.48_, CX/PVA-CDs_0.96_, CX/PVA-CDs_1.44_, and CX/PVA-CDs_1.92_, respectively.

### 2.6. Characterizations

Transmission electron microscopy (TEM) and the high-resolution transmission electron microscopy (HR-TEM) images were obtained using a JEM-2100F at 200 kV. Samples for TEM measurements were prepared by placing a drop of the colloidal solution of CDs on a carbon-coated copper grid and then dried at room temperature. Scanning electron microscopy (SEM, ZEISS Merlin) and atomic force microscopy (AFM, Nanoscope 3a) were used to observe the morphology of the composite films. The sample was mixed with KBr powder at a weight ratio of 1: 100, grinded and pressed into tablet for Fourier transform infrared spectroscopy (FTIR, VERTEX 70, Bruker Corp.). Thermo Gravimetric Analyzer (TGA, TA Q500) was used to measure the thermal properties of samples in N_2_ atmosphere. The temperature was first raised to 100 °C for 5 min to remove physical adsorbed water, and then to 700 °C with a heating rate of 15 °C/min. The ultraviolet visible (UV-VIS) absorption spectrum and transparency of films were measured on UV-VIS Spectrometer (s3100). The fluorescence property was performed on Fluorescence spectrometer (HORIBA Scientific FluoroMax-4). After placing these films in a laboratory with constant temperature and humidity (23 °C and 50% relative humidity) for 24 h, the mechanical properties were carried out on Instron 3342. The size of the tested samples was 15 × 60 mm, and five pieces were tested for each sample. Breaking strength, elongation, and modulus of elasticity were calculated from the average of tensile data.

## 3. Results and Discussion

### 3.1. Surface Morphology of the Composite Films

**Figure 1:**
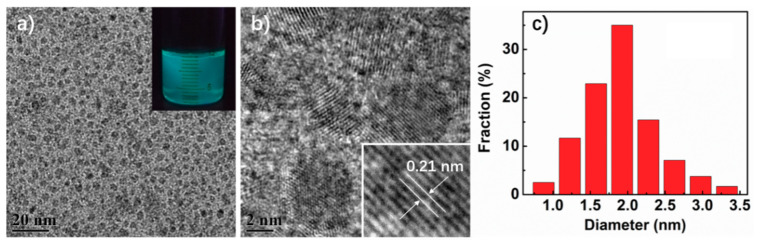
(**a**) TEM image (the inset is the CDs suspension under 365 nm UV); (**b**) HR-TEM image; (**c**) particle size distribution of CDs calculated from TEM image.

The as-prepared xylan-derived CDs exhibit bright blue-green fluorescence under 365 nm UV light (the inset in Figure 1a). The typical TEM image (Figure 1a) shows that CDs are uniform and well dispersed in water, with a narrow size distribution of 1.0–3.5 nm in diameter (Figure 1c). HR-TEM (Figure 1b) reveals clear lattice fringes with a space of 0.21 nm, which corresponds to the (100) diffraction planes of graphite [14].

**Figure 2:**
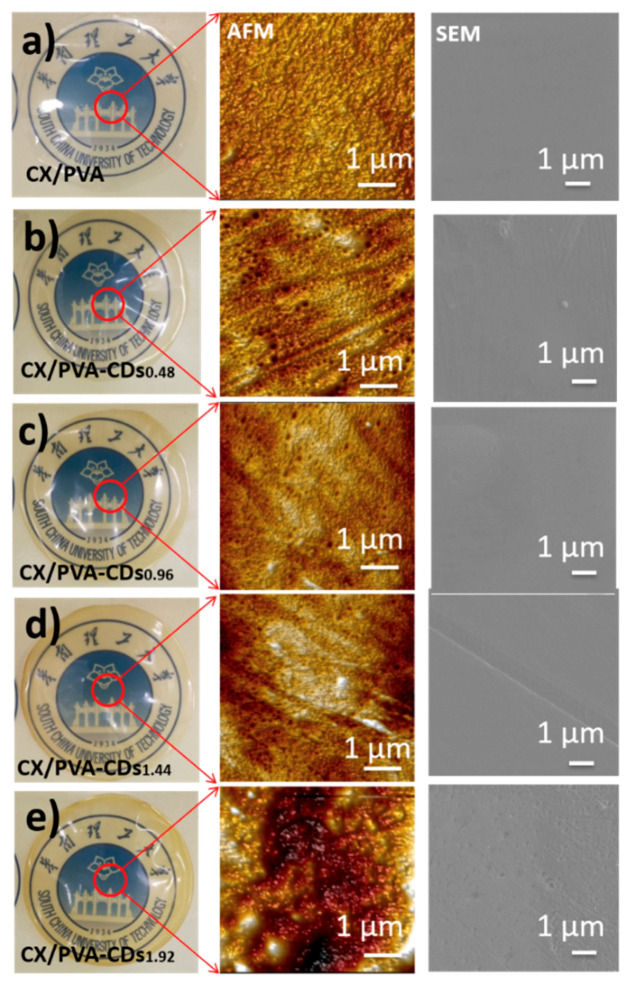
Digital images, atomic force microscopy (AFM) images, and SEM images of CX/PVA and CX/PVA-CDs: (**a**) CX/PVA; (**b**) CX/PVA-CDs_0.48_; (**c**) CX/PVA-CDs_0.96_; (**d**) CX/PVA-CDs_1.44_; (**e**) CX/PVA-CDs_1.92_.

Xylan was carboxymethylated (DS = 0.43, *M*_w_ = 88,541) to improve its water solubility and thus obtain homogeneous CX/CD mixture. PVA was introduced to improve the film-formation performance of CX. As shown in Figure 2a, CX/PVA is transparent and shows smooth surface, indicating good compatibility between CX and PVA; while the nanocomposite films (CX/PVA-CDs, Figure 2b–e) slightly turn yellow with good transparency after adding CDs. AFM image demonstrates that the surface of CX/PVA is compact and uniform, while the surfaces of CX/PVA-CDs become rough and uneven. The increased roughness of the composite film can be attributed to the presence of CDs on the surface of film [28].

### 3.2. FT-IR and TG Analyzations

**Figure 3:**
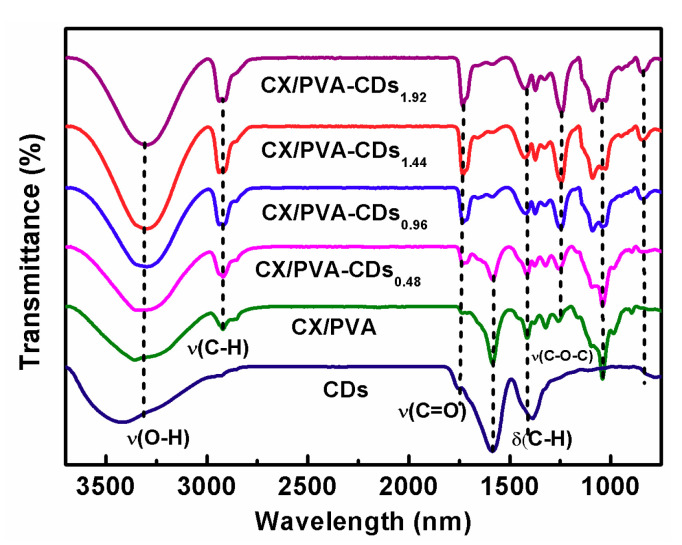
FT-IR spectra of CDs, CX/PVA, and CX/PVA-CDs.

Figure 3 illustrates the FT-IR spectra of CDs, CX/PVA, and CX/PVA-CDs. The peak located at 3300–3400 cm^−1^ corresponds to the O-H absorption of xylan and PVA [31]. The bands at 2927 cm^−1^ and 840 cm^−1^ are attributed to the stretching vibration of –CH_2_. The vibration peak at 840 cm^−1^ is caused by the vibration of C–C in xylan skeleton and the benzene ring skeleton in CDs. In the spectrum of CDs, the peaks at 1760, 1580, 1380 cm^−1^ are assigned to the vibration of C=O, the stretching vibration of aromatic C=C bond and the symmetric deformation vibration of C–H bond in methyl, respectively [18,32]. For CX/PVA, the adsorption at about 1740 cm^−1^ can be ascribed to the stretching vibration of ester functional group C=O [33,34]. The bands at 1080 and 1250 cm^−1^ are caused by symmetric and asymmetric stretching vibration of C–O–C, respectively [35]. The peaks at 1409 and 1580 cm^−1^ confirm the symmetric and asymmetric stretching vibrations of carboxyl ion –CO_2_– in CX, indicating the successful carboxymethylation of xylan. In the spectra of CX/ PVA-CDs, the peak at 1580 cm^−1^ disappears and the peak at 1409 cm^−1^ weakens, which proves that CDs can react with –CO_2_–. With the rise of CDs content, the intensities of bands at 1740, 1250, and 1080 cm^−1^ increase, suggesting that the hydroxyl groups in CDs react with the carboxyl ions in CX to form new ester bond.

**Figure 4:**
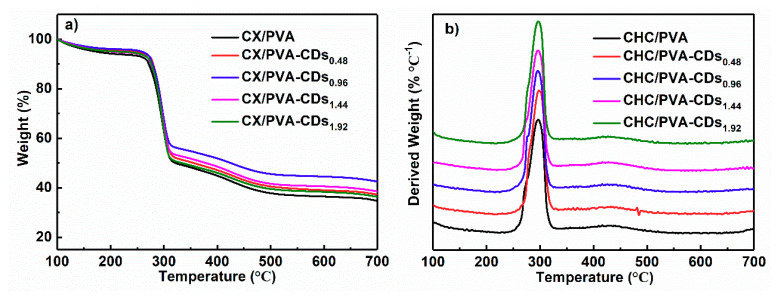
(**a**) TG curves of CX/PVA and CX/PVA-CDs; (**b**) DTG curves of CX/PVA and CX/PVA-CDs.

Figure 4 shows that the mass loss of composite films mainly occurs at 220–325 °C, and the maximum degradation rate happens at about 297 °C. Although the weight retention of nanocomposite film at 700 °C is not closely related to CDs content (may be due to the low CDs loading), the nanocomposite films exhibit higher weight retentions than CX/PVA. As illustrated in Figure 4b, the initial degradation temperatures of CX/PVA, CX/PVA-CDs_0.48_, CX/PVA-CDs_0.96_, CX/PVA-CDs_1.44_, and CX/PVA-CDs_1.92_ are 257, 261, 260, 261, and 260 °C, respectively, indicating that the initial degradation temperatures of CX/PVA-CDs slightly increase, which can be attributed to the chemical bonds between CDs and CX.

### 3.3. Mechanical Performances

**Figure 5:**
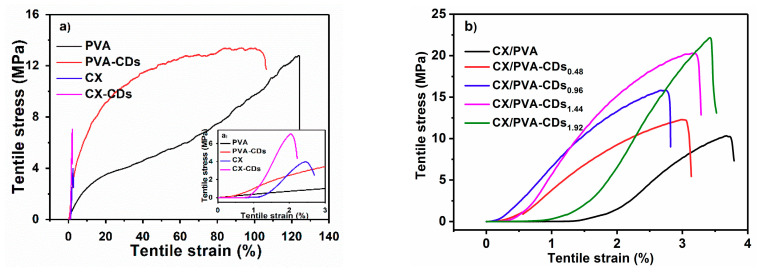
(**a**) Stress-strain curves of CX, CX-CDs, PVA, and PVA-CDs; (**b**) stress-strain curves of CX/PVA and CX/PVA-CDs.

Figure 5 and Table 1 show the mechanical properties of the as-prepared films. Pure PVA film presents high elongation at break (more than 100%) and moderate tensile strength (12.81 MPa, Figure 5a), while pure CX film shows low elongation (2.45%) and low stress (3.97 MPa, Figure 5a). However, the tensile strength of CX/PVA composite film is significantly improved due to better film formation, with an elongation of 3.67% and stress of 10.34 MPa (Figure 5b). As compared with CX, the tensile strength of CX-CDs_1.92_ nearly doubles (7.06 MPa, Figure 5a), while that of PVA-CD_1.92_ only slightly increases (comparing with pure PVA film) (Figure 5a). The difference in the increase of tensile strength can be attributed to the chemical interaction between CDs and CX. The breaking elongations of CX-CDs_1.92_ and PVA-CDs_1.92_ slightly decrease. The films undergo two tensile stages in the test. The first stage occurs within 2% tensile strain, where tensile stress increases slowly with strain. In the second stage, the composite film is further stretched and the stress rapidly increases with strain. Non-necking can be observed in the second stage, indicating the homogeneous distribution of CDs in the film. As shown in Figure 5b, the addition of CDs significantly enhances the tensile property of CX/PVA-CDs. The tensile strengths of CX/PVA-CDs_0.48_, CX/PVA-CDs_0.96_, CX/PVA-CDs_1.44_, and CX/PVA-CDs_1.92_ are 12.29, 15.83, 20.28, and 22.16 MPa, respectively. Especially, with 1.92% CDs, a remarkable increment of 114.3% in tensile strength can be obtained. The elastic modulus of the nanocomposite film also significantly increases with CDs content. For example, the elastic modulus of CX/PVA-CDs_1.92_ (1363.11 MPa) is increased by 90.7% as compared with that of CX/PVA (714.70 MPa). The increase of elastic modulus can be ascribed to stronger interaction between CDs and CX, as indicated in Figure 3) at higher CDs loading. The elongation at break doesn’t change significantly. Therefore, CDs can effectively enhance the mechanical strength of the composite films due to its interaction with CX.

### 3.4. Optical Performance of CX/PVA-CDs Composite Films

**Figure 6:**
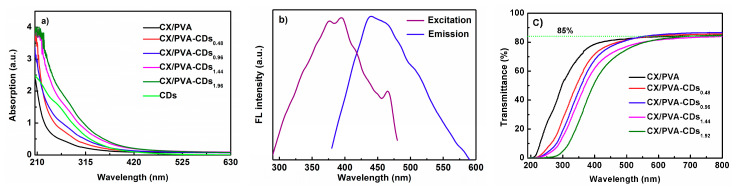
(**a**) Absorption spectra of CX/PVA and CX/PVA-CDs films; (**b**) fluorescence spectra of CX/PVA-CDs_1.92_; (**c**) transmittance spectra of CX/PVA and CX/PVA-CDs films.

Figure 6a demonstrates that CX/PVA film shows no significant absorption at light waves higher than 250 nm, while CX/PVA-CDs films have a continuous absorption in the UV region, especially at 260–305 nm, which echoes the absorption spectrum of CDs. The absorption intensity of CX/PVA-CDs composite films in the UV region increases with CDs content. Therefore, CX/PVA-CDs composite films can be used as light conversion films. Figure 6b reveals the fluorescence spectra of CX/PVA-CDs_1.92_ composite film. It has a strong emission peak at 440 nm under 360 nm excitation light, and an excitation peak at 376 nm under 540 nm emission light. The results demonstrate that CX/PVA-CDs_1.92_shows the characteristics of UV excitation and blue light emission, which indicates the potential application of CX/PVA-CDs_1.92_ in the light conversion.

Figure 6c illustrates that the light transmittance of CX/PVA film without CDs is high at the UV region of 200–380 nm, which is reduced after adding CDs, indicating that CX/PVA-CDs composite film can block UV. At the light wave of higher than 600 nm, the light transmittances of CX/PVA-CDs and CX/PVA are similar (~85%), suggesting that light beneficial to crops is not blocked. Therefore, CX/PVA-CDs composite film is expected to be used in agricultural planting and food packaging to block UV.

## 4. Conclusions

Xylan-based nanocomposite films with excellent light conversion performances can be prepared through solution mixing method by using xylan-derived CDs as both light conversion regents and nano enhancements. CDs have a good compatibility with matrix and the composite film has uniform structure. The nanocomposite film has better thermal stability. Meanwhile, chemical interaction is formed between carboxymethyl xylan and CDs, which significantly enhances the mechanical strength of the nanocomposite films. Especially, with only 1.92% CDs, the tensile strength and elastic modulus of the nanocomposite film are increased by 114.3% and 90.7%, respectively. The nanocomposite film shows UV excitation and excellent optical transmittance, and can effectively convert UV to blue light. These properties render the potential applications of the nanocomposite film in food packaging and agricultural planting.

## Figures and Tables

**Table 1:** The breaking strength, elongation, and modulus of CX, PVA, CX/PVA, CX-CDs, PVA-CDs, and CX/PVA-CDs films.

Samples	Breaking Strength (MPa)	Breaking Elongation (%)	Modulus of Elasticity (MPa)
CX	3.97	2.45	416.45
CX-CDs	7.06	2.04	719.31
PVA	12.81	124.22	24.19
PVA-CDs	13.11	103.6	35.07
CX/PVA	10.34	3.67	714.70
CX/PVA-CDs_0.48_	12.29	2.99	690.33
CX/PVA-CDs_0.96_	15.83	2.75	869.12
CX/PVA-CDs_1.44_	20.28	3.15	1204.75
CX/PVA-CDs_1.92_	22.16	3.42	1363.11
